# Benefits, challenges and contributors to the introduction of new hospital-based outpatient clinic pharmacist positions

**DOI:** 10.1016/j.rcsop.2022.100119

**Published:** 2022-02-26

**Authors:** Centaine L. Snoswell, Amelia R. Cossart, Bernadette Chevalier, Michael Barras

**Affiliations:** a Pharmacy Department, Princess Alexandra Hospital, Brisbane, Australia; b Centre for Health Services Research, The University of Queensland, Brisbane, Australia; c School of Pharmacy, The University of Queensland, Brisbane, Australia; d Department of Family Medicine, University of Alberta, Edmonton, Canada

**Keywords:** Outpatient, Pharmacists, Qualitative research, Interviews, Specialist clinics

## Abstract

**Background:**

Pharmacists working within interprofessional teams in the outpatient setting are well placed to address medication-related problems before and after hospital admission. Therefore, exploration of these roles is warranted.

**Objective(s):**

To explore pharmacists' and other health professionals' perspectives of the impact of pharmacists working within interprofessional teams in outpatient clinics. Furthermore, we endeavoured to identify both the challenges and contributors to success with the introduction of pharmacists into these settings.

**Methods:**

This qualitative study involved semi-structured interviews with both hospital outpatient clinic pharmacists and other clinic health professionals to gain an in-depth understanding of how the introduction of pharmacists into clinics impacted clinic processes, patient care, and relationships with other health professionals. Participants were recruited from the outpatient clinics who had recently added a pharmacist to their service. Participants involved in setting up the roles were invited to participate in a voluntary interview, the transcripts from which were analysed into themes and sub-themes using an inductive and deductive approach.

**Results:**

A total of 34 staff were interviewed of which 68% were female and 74% were aged between 31 and 50 years. The cohort included 16 outpatient pharmacists, nine pharmacist team leaders, five clinic nurses and four clinic doctors (specialist consultant or registrar). Three overall themes were identified: the benefits, the contributors, and the challenges of introducing clinical pharmacy services to outpatient clinics. When establishing a clinic role, pharmacists' awareness, adaptability, and strong communication were shown to be key traits to building rapport and trustworthiness with the established clinic team.

**Conclusions:**

When pharmacists are integrated into multidisciplinary outpatient clinics they and their colleagues believe that they provide benefits to the patients and the clinics. Decision makers need to be cognizant of factors that contribute to, as well as those that impede, the successful implementation of outpatient pharmacist roles.

## 1 Introduction

Medicines form a critical part of patient care and management, however, if used incorrectly or in error, medicines can cause serious harm.^1^^,^^2^ Roughead and researchers showed in 2004 that 90% of community patients at risk of medication misadventure experienced one or more medication-related problems.^2^ These problems can lead to medication harm which can have detrimental effects to patients such as delayed recovery or rehospitalisation.^3^ In addition, medication-related harms are costly to healthcare systems. It is estimated that these costs represent approximately 1% of the global health expenditure or US$42 billion per annum.^1^ Pharmacists have been identified as the health professionals with the expertise to reduce medication-related harm, especially those occurring at transitions of patient care, for example tertiary-primary care, where there is an increase in the incidence of medication errors.^2^

Healthcare provided in Australian hospital outpatient setting is commonly used as an intermediary link between hospital and community care.^4^ For example, many patients who are discharged from hospital are asked to return and be reviewed by the treating medical team within the outpatient setting. These patients often have complex medical and medication needs that require re-evaluation 2 to 6 weeks post-discharge to evaluate whether new therapies are efficacious and well tolerated or require adjustment. Pharmacists working within interprofessional teams practising in the outpatient setting are well placed to evaluate patient progress and address any medication-related problems. Pharmacists can also facilitate the timely dissemination of medication-related information through effective communication and engagement with patients and their interprofessional colleagues; both locally and in primary care (e.g. general practitioners (GPs) or community pharmacists).^5^^,^^6^ These contributions by pharmacists are particularly relevant in the increasing complexity of care associated with the rise in chronic disease, an ageing population, and associated medicine issues such as polypharmacy.^7^

While pharmacists have been acknowledged to be integral to patient management during hospital admission, their roles are not well-established within the interprofessional team in outpatient clinics in Australia.^5^^,^^8^ To date, the majority of literature examining pharmacists in this setting has focused on discipline specific roles such as cancer clinics or cardiovascular care, and the measurement of discipline-specific outcomes such as hypertension and anticoagulation.^9^, ^10^, ^11^, ^12^, ^13^ For example, a single-discipline case study by Collier and Baker (2014) reported the impact of having introduced a pharmacist into an outpatient clinic for veterans with poorly controlled type 2 diabetes.^14^ Interventions made by the pharmacist were shown to increase satisfaction with patient-clinician relationships and significantly improve insulin management and HbA1C control (*p* < 0.0001), through supported individualised patient healthcare.^14^ This finding was mirrored by an Australian study that showed including an outpatient pharmacist into a multi-disciplinary team, caring for patients with chronic liver disease, resulted in better identification of medication-related problems, and a greater proportion of the high risk problems being resolved (versus low or medium risk problems, *p* < 0.0001).^6^ These studies and reviews suggest that the involvement of pharmacists in patients’ medication management post-discharge fosters patient-centred care, enables patients to self-manage their medications at home, and reduces overall cost due to a reduction in acute hospital readmissions.^15^, ^16^, ^17^

Based on these promising patient outcomes associated with the introduction of pharmacists to multidisciplinary and discipline-specific hospital outpatient teams (i.e. hepatology or cancer), and the growing need for effective medication management of patients with complex chronic diseases, there exists a clear role for further integration of pharmacists into hospital-based clinics more broadly. Current research evaluating this concept has not included a comprehensive assessment of the pharmacists' collaborative impact, but instead focuses on reducing adverse medication events or improving patient outcomes. This research was novel as it used a qualitative methodology to evaluate the impact of introducing outpatient pharmacists into 14 clinics that have not previously had pharmacist involvement. This paper will add to existing work in pharmacy practice by examining the role of pharmacists in the hospital outpatient setting. The intent is to draw valuable information from individuals experiencing the roles to better understand pharmacists' and their colleagues' perspective of having pharmacists integrated into outpatient clinic teams.

## 2 Objectives

To explore pharmacists' and other health professionals' perspectives of the impact of having pharmacists working within interprofessional teams in hospital outpatient clinics, as well as the challenges and contributors to the successful introduction of pharmacists into these settings.

## 3 Method

This study involved semi-structured individual interviews with outpatient pharmacists and clinic health professionals to obtain rich detail and an in-depth understanding of how the introduction of pharmacists into outpatient clinics impacted clinic processes, patient care, and relationships with other healthcare professionals. Ethics approval for this study was obtained from the Metro South Human Research Ethics Committee (approval HREC/2020/QMS/61133). Verbal or written consent was provided by all participating pharmacists and healthcare professionals from hospital outpatient clinics. Results have been reported in line with the consolidated criteria for reporting qualitative studies (COREQ) framework.^18^

### 3.1 Setting

Participants were recruited from the outpatient clinics receiving new clinical pharmacy services within the Princess Alexandra Hospital (PAH), Brisbane, Australia. The PAH is a large, 900-bed, public, metropolitan, adult, tertiary referral hospital and receives approximately 110,000 patients annually. In the 2019–2020 financial year, the PAH Business Executive used activity based funding opportunities to fund the addition of pharmacists to 14 outpatient clinics (Table 1) resulting in a total of 10.2 additional full time equivalent pharmacists (16 pharmacists working either part-time or full time).^40^, ^41^, ^42^ This research focuses on these pharmacists' roles which were expected to become integral to the outpatient team environment and foster interprofessional collaboration.

**Table 1:** Outpatient clinics, role full-time equivalent (FTE) and service description.

Clinic	FTE	Description of clinic (i.e. types of patients)
Brain injury rehabilitation unit (BIRU)	1	Patients with history of Brain Injuries in both the outpatient and inpatient setting. The pharmacist works within the multi-disciplinary Day Hospital which is the only ambulatory service specialising in acute brain injury in Queensland, Australia.
Kidney replacement therapy	0.4	Patients on kidney replacement therapies including incentre and home haemodialysis and home peritoneal dialysis patients.
Cancer services	2	Patients requiring a clinical verification of cancer therapy protocols prescribed for inpatients and outpatients.
Cardiac pre-admission	0.1	Medication history documentation & pre-operative medication planning for patients undergoing cardio-thoracic surgery.
Endoscopy pre-assessment	0.2	Review of pre-endoscopy patients in an outpatient setting, ensuring comprehensive medication histories and compliance assessments.
Gastroenterology services	1	Gastroenterology and hepatology outpatients. Pharmacist provides specialist inpatient care, research assistance, and accepts referrals for community management.
Geriatric Emergency Department Initiative (GEDI)	0.4	Pharmacist review of frail, elderly patients that have been referred to the GEDI clinic for geriatrician and pharmacist review within 7 days following a recent hospital presentation.
Immunology services	0.5	Highly complex patients with immune system disorders such as allergy, immunodeficiency or autoimmune conditions requiring specialist immunology care.
Mental health older adults	0.5	Patients aged >65 years with a Serious Mental Illness (SMI) and co-existing medical co-morbidities who are under the care of the Older Adults Mental Health Community Team.
Mental health community outpatients	1	Patients aged 18–65 years with SMI who are under the care of adult community mental health services (Mood, Psychosis, Rehabilitation, Early Psychosis and Clozapine Clinics).
Ophthalmology services	1	Ophthalmology pharmacist provides inpatient and outpatient care. Provides expert knowledge, operational services for the multi-disciplinary team, and district-wide collaboration.
Kidney services	0.5	Outpatients with Chronic Kidney Disease requiring kidney specialist follow up.
Kidney transplant services	1	Outpatients who have received a kidney transplant and require ongoing care by kidney transplant unit (includes both acute and chronic patients). Patients who are undertaking work-up to assess their suitability for kidney transplant.
Security services	0.6	Medication history documentation and pre-operative medication planning and communication to stakeholders for prisoners undergoing any surgery in the hospital. Outpatient service to ensure continuity of care/treatment pathway communication/access to restricted medications.

### 3.2 Participant recruitment

All hospital outpatient pharmacists, their team leaders, and other senior management in the Pharmacy Department involved in setting up the roles were invited via email to participate in a voluntary interview. A snowball strategy was used for further recruitment whereby participating pharmacists were asked to invite a non-pharmacist clinician from their clinic to participate to ensure different health professional perspectives were captured. There was no interaction with patients during any stage of this research. There was no compensation or incentives provided to participants involved in this study. Recruitment ceased once data saturation was reached.

### 3.3 Data collection

Prior to the interview, participants returned their signed consent forms and completed demographics questionnaire. Verbal consent was collected for individuals who were unable to return the forms in advance of the interview. Interviews occurred via phone, videoconference, or in-person depending on the availability of the participant and the current COVID-19 restrictions. For consistency, interviews were conducted by one interviewer (MT), independent of the research team and hospital. An interview guide of questions and prompts was followed for each interview (Appendix A). The interviews were audio-recorded and transcribed verbatim for analysis by one interviewer (AC).

### 3.4 Data analysis

Common concepts within transcripts were initially coded, and then grouped into sub-themes and themes. NVIVO 12© software was used to organise the codes. Each transcription was coded by one member of the research team (AC), with themes and sub-themes derived using an inductive and deductive thematic analysis approach.^19^ Reliability testing of codes and themes was conducted by having a second researcher (BC) independently generate themes and sub-themes based on a random sample of 5 transcripts (15%). Peer-debriefing was conducted to reach consensus regarding overarching themes and subthemes (AC, BC, CS). Exemplars were chosen to illustrate each theme and subtheme, and to support transparency and trustworthiness of reporting.^18^

### 3.5 Trustworthiness and rigor of method

To ensure that research is rigorous and trustworthy, it is critical that the methodology be described in detail. Trustworthiness requires the research to be credible, transferable, dependable, and confirmable.^20^^,^^21^ Credibility in this study meant that the authors involved in the study were experienced clinical pharmacists with hospital pharmacy expertise, of which two (CS, MB) were employed at the study site. These pharmacists understood the role of pharmacists within an interprofessional team and were knowledgeable about the work environment of the PAH in which the study participants practiced. However, these researchers were not involved in clinical pharmacy activities in outpatient clinics at the PAH at the time of the study. In addition, authors employed by the PAH did not review the interview transcripts or provide input until after the themes had been refined.

Transferability can be challenging; however, this study involved the introduction of new clinical pharmacy services to a broad range of outpatient clinics. In fact, 16 outpatient pharmacists were introduced to 14 unique clinics. Perspectives of all pharmacists introduced to these clinics were sought through interviews. Additionally, the interviews with non-pharmacist health professionals were conducted across a range of outpatient clinics to reduce the bias from a single clinical discipline and setting.

Dependability was ensured by employing independent (non-hospital team member) researchers to conduct the interviews (MT) and analyse the transcripts (AC). Field notes were taken throughout the interviews and a record of all changes to methodological processes was documented. Confirmability was established by having another independent researcher (BC) with hospital pharmacy experience code a random selection of interview transcripts to ensure that coding reflected consistent and appropriate interpretation. Areas of disagreement were resolved through consensus.

### 3.6 Reflexivity

Having only practiced in the community pharmacy setting, AC felt she could bring a unique and less biased viewpoint when examining the impact of pharmacists in the outpatient clinic setting. Nonetheless, AC acknowledges that she has strong opinions about best practice and the current underutilisation of pharmacists across the healthcare setting, which may have influenced her perception and interpretation of the data. However, participants were aware that the interviewer (MT), was not associated with the research team or healthcare, which meant that discussions were guided by the interviewee, and it was less likely the participants would have felt influenced to respond in a particular manner.

## 4 Results and discussion

A total of 34 hospital staff were interviewed from the 14 hospital outpatient clinics that added at least one pharmacist to their team in mid-2019. A brief description of the clinic and number of pharmacists per clinic are shown in Table 1. Most of the interviewees were female (68%) and 74% were between 31 and 50 years of age. Those interviewed included 16 outpatient pharmacists, nine pharmacy team leaders, five clinic nurses and four clinic doctors (specialist consultant or registrar) (Table 2). The length of time for interviews ranged from 30 min to 1 h.

**Table 2:** Participant demographics.

Position	Categories	Count (%)
Gender	Female	23 (68)
Age (years)	21–30	6 (18)
	31–50	25 (74)
	>50	3 (9)
Health professional position	Pharmacist	16 (47)
	Pharmacist and team leader	9 (26)
	Specialist consultant or registrar	4 (12)
	Nurse (including clinical nurse, registered nurse or telehealth nurse navigator)	5 (15)
Highest level of education	Bachelor	13 (38)
	Graduate certificate	8 (24)
	Graduate Diploma	6 (18)
	Masters	6 (18)
	PhD	1 (3)
Years practicing as a pharmacist or other health professional	Less than 1 year	1 (3)
	1–5 years	8 (24)
	6–10 years	8 (24)
	11–15 years	9 (26)
	16–20 years	5 (15)
	21+ years	3 (9)
Previous experience as a clinical pharmacist in a patient care area	Less than 1 year	1 (3)
	1–5 years	14 (41)
	6–10 years	9 (26)
	11–15 years	5 (15)
	16–20 years	3 (9)
	21+ years	2 (6)
Area of specialty	Rehabilitation	3 (9)
	Medical	9 (26)
	Surgical	8 (23)
	Renal	4 (12)
	Cancer	5 (15)
	Other (emergency, immunology, mental health, misc.)	5 (15)

Three overall themes were identified and include the benefits, the contributors, and the challenges to introducing clinical pharmacy services to outpatient clinics. These themes, subthemes and related codes are listed in Table 3, and described in detail below with exemplars.

**Table 3:** Themes, subthemes and codes.

Theme	Subtheme	Code
Benefits	Patient specific	Continuity of care/preventing communication breakdown
		Empowerment
		Improved medication management
		Patient safety
	Clinic specific	Multi-disciplinary collaboration
		Improved workflow/workload management
	Healthcare system	Improved use of healthcare resources
Contributors to success	Building effective interprofessional relationships	Teamwork
		Supporting non-pharmacist clinic staff
		Interprofessional communication
	System wide support	Ongoing funding
	Pharmacist attributes	Adaptability
Challenges	Pharmacist specific	Pressures to capture/measure clinical activities
	Clinic specific	Scheduling challenges
		Space/“real estate”
	Multi-disciplinary team breakdown	Lack of communication
		Working apart from clinicians
		Lack of role clarity
		Tunnel vision healthcare
	Patient specific	Patient fatigue

## 5 Theme 1: Benefits associated with the introduction of clinical pharmacy services

### 5.1 Subtheme 1: Patient specific

#### 5.1.1 Continuity of care/preventing communication breakdown

Pharmacists described how they play an important role in the continuity of care in patients' transition from hospital to primary care ensuring medication information is effectively relayed to ‘non-hospital’ members of the healthcare team such as GPs and community pharmacists.

*“So - communicating to the patient, as well as communicating to, you know, potentially community pharmacies. … You know, providing some advice about medications to GPs or what has been potentially prescribed here…”*

(P08)

Without this timely transfer of information, pharmacists recognised that there is often a breakdown in communication, as all parties have not been informed of, and understand the rationale for changes in medication therapy, particularly when long-term medications are ceased in hospital. The World Health Organisation emphasises the need for strong communication skills, stating that that the key to successful transitional care, and reduced medication-related errors, is effective communication.^1^ Pharmacists play an important role in transitional processes^22^^,^^23^ and have been shown to reduce the number of medication errors, adverse drug events and hospital re-presentation.^15^^,^^16^^,^^24^

#### 5.1.2 Empowerment

Pharmacists described how they want patients to play an active role in their own healthcare which improves patient safety.

*“You're wanting to empower people to make decisions themselves. So- you want a situation where it's a collaborative sharing of information, so, everyone is going - to the best outcome for the patient”*

(P03)

Pharmacists spoke about how they aimed to take a holistic approach to healthcare, how doing so better facilitates patient engagement, increases patients' agency, and empowers patients to play an active role in their own medication management. One pharmacist noted this change in patient engagement and reported how some patients now:

*“…refuse to take a medication because they know they've been instructed not to ... So - they are in charge of the healthcare now... where they are in charge of the process and they know what's happening.”*

(P06)

Pharmacists spoke about how they were willing to go that ‘extra mile’ to assist patients in navigating the complex, confusing and often overwhelming healthcare system for patients. For example, pharmacists actively referred patients as the most appropriate member of the healthcare team to look after the patient's needs. Study pharmacists described how they empowered patients by increasing their knowledge of how to best utilise the healthcare system, and reflected that this empowerment would contribute to a positive patient journey. Health locus of control research^25^ supports this belief and has identified that powerful others (e.g. the healthcare team) influence positive health behaviours by patients.^26^

#### 5.1.3 Improved medication management

Pharmacists explained how they support patient self-management by improving medication-related health literacy, simplifying medication therapy, encouraging and supporting medication taking behaviours that foster adherence, deprescribing unnecessary medications, and identifying and resolving medication-related problems.

“…*things that we might think might not have a big clinical impact, might have a big day- to- day impact... If constipation is a really big problem for them and getting a phosphate binder changed for that patient to something that's less constipating... it's not, that's not a life-saving intervention, but on a day-to-day basis that can have a really big impact on that patient's quality of life.*”

(P08)

“*…it actually scares me to think how the medication management was done [before]… especially with the supply of some of the medications. [The pharmacist helps to support] patient safety, ensuring patient safety and continuity of care*”

(P31)

Other research has supported that medication changes made by pharmacists may not be clinically complex; however, simple changes can still have a profound impact.^27^ In addition, by focusing on the individual patient and their specific needs,^15^ pharmacists build trust and strengthen their pharmacist-patient relationships.^28^

#### 5.1.4 Patient safety

While some medication harm is not preventable,^2^^,^^3^^,^^5^^,^^29^ the current high rates of hospital readmission globally are in part attributed to avoidable medication errors at transitions of care.^8^^,^^30^ However, when robust systems are in place, and healthcare teams work collaboratively to drive patient-centred healthcare, reductions in medication errors have been demonstrated.^1^^,^^7^^,^^22^

Pharmacists cited examples of how they felt their professional contribution to the clinic reduces medication errors which in turn, improves patient safety. Pharmacists explained that consultations with patients allows them to:

*“…pick up on medication interactions, new medications, new supplies that they shouldn't have had or supplies that they've run out of. Problems with their compliance…”*

(P20)

These pharmacist interventions have the potential to positively benefit a patient's overall health and state of well-being. This belief was supported by clinicians (nurses, doctors and others) who said that by having an up-to-date medication history, they had:

“…*a starting point… when we're doing that patient's admission [and] from a transfer of care, in between outpatients and inpatients, that's probably more streamlined. And safer from a medication safety point of view - that we have access to up-to-date data…”*

(P25)

“*pharmacist that was available would be able to go into a more in-depth explanation about the patient medications and why we need to stop certain medications before surgery, as well as just giving them general education*”

(P07)

### 5.2 Subtheme 2 Clinic specific benefits

#### 5.2.1 Multi-disciplinary collaboration

Study pharmacists described how health professionals working in a team environment improved the clinic's efficiency by streamlining processes to reduce duplication of effort and by improving the communication handover procedures. .

This does not always occur, and the fragmented approach to communication between health professionals at transitions of care can have negative effects on health outcomes, increasing the financial burden on the healthcare system.^4^

Pharmacists regularly reflected on their skill set and endeavoured to refine their knowledge base and learn from other team members. This desire for shared learning was echoed by the other health professionals interviewed who felt that working closely in their team-based setting enabled interprofessional learning opportunities. Interviewees believed that interprofessional collaboration fostered trust and allowed each discipline to focus on their own area of expertise. Nursing staff in the renal clinic described how they believed the pharmacist to be indispensable:

*“…Because they are a very complex group of patients with heavy, significant co-morbidities. So - the role of the pharmacist is, you know essential in, to ensure that a, b, and c happens. You know, in regard to appropriateness of the care we provide. And it's always having that eye for detail that each specialist has…”*

(P16)

The findings were supported by Chevalier et al., who similarly reported that the majority of nurses and physicians survey respondents agreed that having a pharmacist on their healthcare team to help manage medication issues allowed them to feel more confident and better able to concentrate on their own professional roles.^31^

#### 5.2.2 Improved workflow/workload management

Having a pharmacist within the clinic's healthcare team was believed to result in better physician preparedness for patient consultation, and a shared distribution of the clinical workload. This change may ultimately benefit the patient, as a clinician who is updated and more knowledgeable at the time of consultations could reduce the risk of medication errors and avoid duplication of work.^15^ The following exemplar describes the benefits of having a team work together to create and support a patient care plan:

*“...with the pharmacist added onto the team, the doctor can actually get more advice from a pharmacist and then give to the patient and definitely after the follow up, the pharmacist can help with that and then they can give more clear advice to the patient”*

(P24)

### 5.3 Subtheme 3 Benefits to the Healthcare system

*Improved use of healthcare resources reduces failure to attend rates.* Pharmacists' roles reduce the cancellation of hospital appointments and procedures. A doctor explains how the pharmacist will:

*“...flag all the changes or things that we need to do before the procedure and [as a result they've] had very few failure-to-attend events.”*

(P06)

The resultant reduction in readmissions, cancellations, and failure-to-attend events, lessens the unnecessary use of healthcare resources and the associated economic impact.^6^^,^^23^^,^^29^

## 6 Theme 2: contributors to successful implementation of clinical pharmacy services

### 6.1 Subtheme 1 Building effective interprofessional relationships

#### 6.1.1 Teamwork

Both pharmacists and other health professionals indicated that pharmacists who became integrated within the healthcare team were better able to support and contribute to shared decision making, as illustrated in the following comment: *“…the better the pharmacist integrates in the team, the more successful they are affecting changes. … if you're able to not only provide a service but integrate again with what the clinic overall is trying to achieve and be directly involved with the decisions made” (P14)*.

Clinic health professionals commented on how this team-based approach with the inclusion of pharmacists' expertise had a multitude of benefits for patient care. Examples such as recommendations for medication changes, comprehensive medication reviews, prescription and blood work follow-ups were felt to contribute to better patient outcomes and a positive patient health journey.

One pharmacist provided examples of how they had liaised with multiple health professionals: *“…initially with the GP, because this patient had not yet been scheduled to see a doctor [hospital clinician]…Gave some recommendations and …prompted for a medical review as well….The GP implemented some of our recommendations and they had helped that patient - reducing the dose of night-time Targin®, and yeah, he was getting less nightmares hallucinations, and [encouraging appointments] with physio and OT…”* (P05).

#### 6.1.2 Supporting non-pharmacist clinic staff

Pharmacists felt that the development of positive relationships with clinic staff fostered trust in their clinical judgement and decision making which in turn has led to further openness to collaborate. It appears obvious from interviewee comments that the pharmacists who built positive relationships with other members of the multi-disciplinary team were able to nurture a supportive and collaborative environment in which they could share ideas and enact change and be willing to make changes to the services as needed.

*“...I guess support from the [clinician] because…Yes, I can make a good recommendation. But if I don't have the [clinician] on board, they may not want to make changes. Then it kind of negates the...recommendation itself. So having that, I guess, that relationship and having that trust that they're willing to take the advice on board.”*

(P21)

“*So it's quite interesting for our particular clinic in that we went in with one intention, and that was a pharmacist in an allied health clinic, so physiotherapy clinic to review patients medications and issues with pain medications. But we found that the business wasn't there as much as we needed. So we actually had to expand and branch out as the clinic went along to include other patient cohorts.*”

(P17)

#### 6.1.3 Interprofessional communication

Pharmacists believed their professional role within the healthcare team centres on effective communication, which in turn supports efficiency of care delivery and patient safety. Pharmacists described how they communicate decisions made by one specialist to another, how they engage in structured handovers during clinic conference meetings as well as the ways in which they liaise with patients' primary care clinicians to ensure effective transitions of care.

*“...if the psychiatrist makes any changes to medications, then I'll call the GP and I'll call the pharmacist directly and make sure that change is done straight away”*

(P21)

This finding is also reflected by Jorgensen, who showed that trust and active two-way communication was key to multi-disciplinary collaboration.^32^ Antony et al. concluded that maintaining active communication channels between health professionals is integral to successful relationship building.^33^

### 6.2 Subtheme 2 System wide support

#### 6.2.1 Ongoing funding and support

Establishing consistent and ongoing funding and support was one of the key enablers identified for the continuation of the outpatient pharmacist clinic positions.

Pharmacists also describe how support from administrative officers and dispense technicians is critical to the workflow through the clinic which affects potential funding continuation.

*“And also just having that really strong admin staff. I can't imagine myself being able to, you know, manage the workload as I had, if I needed to chase up my own patients because again, my patients are very difficult to chase up. But having yeah really strong admin staff there to make yeah allies a lot easier is quite important as well.”*

(P05)

### 6.3 Subtheme 3 Pharmacist attributes

#### 6.3.1 Adaptability

Being introduced into a new environment that did not previously have clinical pharmacy services made it challenging for pharmacists to establish themselves within the healthcare team as the medication expert. For example, pharmacists are trained to question the decisions of colleagues when they perceive those decisions to not be in the best interest of patients. However, study pharmacists explained how they were tentative, acutely aware of the roles and responsibilities of other team members, and cautious of how their role may be perceived. As a result, initial communication was carefully conducted to build trust and rapport with clinic staff. Furthermore, pharmacists explained how they are always learning and refining their skillset based on non-verbal and verbal feedback so that they can adapt to be active contributors to the team.

Having this self-awareness around adaptability seemed to be a key attribute for a pharmacist's successful integration into a clinic team and with their interaction with patients, as described by a pharmacist in the exemplar below:

*“Clinical progress is not the key to clinic success. It's much more than that. It is the ability for a person to adapt to new and unpredictable situations. And then go and tailor the communication to the patient. So each patient, you get to see will have a different requirement for you as a pharmacist to get the information out of them”*

(P03)

## 7 Theme 3: Challenges associated with introduction of clinical pharmacy services

### 7.1 Subtheme 1 Pharmacist specific

#### 7.1.1 Pressure to capture and measure clinical activities

Funding for these roles is dependent on activity targets, therefore the pharmacists were required to conduct sufficient numbers of patient consultations in order to ensure the sustainability of their role. This concern for ongoing funding was expressed by many pharmacists:

*“ …ensuring the funding keeps going. I guess because if there's no funding then we can't be there…”*

(P21)

Clinical activity targets (number and type of consults) were set for each pharmacist based on estimated activity. However, as future service delivery was not fully understood, the initial targets were not always proportional to the staffing model of the clinic. One outpatient pharmacist explained:

*“...reviewing patients [conducting a consult] is … actually a very small part, it's only one part of the, the role itself. So a lot of [the role] is actually spent on communication, documenting reports, sending letters to the GP, and follow up.”*

(P21)

Consequently, pharmacists felt it was difficult to balance tasks they believed were more critical to patient care to the required clinical activity targets measured to validate their professional work for ongoing funding. Often, this mismatch between what needs to be done and what needs to be measured resulted in pharmacists following up with patients outside their rostered clinic time, and a sense of frustration:

*“I think they [the Executives] need to consider patient care more. They need to consider like, you know, why we do this. And that it's not just about money. Um, you know, we can't compromise patient lives and cause more issues by not considering the bigger picture. And so I really think that, you know, across the board, there's a lot of things that are so dependent on finance. We just miss the important things”*

(P04)

It is worthwhile noting that these competing job requirements contribute to workplace stress for pharmacists and may negatively impact both patient care and pharmacist well-being as described by other researchers.^34^, ^35^, ^36^ These workloads associated with balancing documentation and direct patient care issues are not sustainable and need to be addressed by decision makers.

### 7.2 Subtheme 2 Clinic specific

#### 7.2.1 Scheduling challenges

The current funding model requires each health professional to book individual appointments with patients that cannot overlap which means that patients or staff are often waiting when appointments run shorter or longer than expected. To reduce wait time, pharmacists often see patients opportunistically while they wait for other health professionals and appointments are booked retrospectively. This is sometimes perceived to alter/interrupt the flow of clinic by other health professionals. Some pharmacists also believed that patients feel their clinic time is unnecessarily extended leading to patient frustration and appointment fatigue making it more difficult for the pharmacist to have a meaningful and productive consultation with the patient about their medications.

*“So I think that was a difficulty at the start, just trying to slot myself in. Particularly given my patient cohort, they get very fatigued, very easily. And that's something I'm also quite mindful of when I see them”*

(P05)

Furthermore, the optimum workflow for the team would be for the pharmacist to see the patient first, identify medication problems and then consult with the clinician to action these problems. However, lack of appointment scheduling prevents this flow in some clinics.

#### 7.2.2 Space/“real-estate”

The lack of designated space for pharmacists to consult with patients in a private setting seems to be detrimental to both the workflow through the clinic and the development of patient-pharmacist relationships. A doctor described how she:

*“…feels sorry for [the pharmacist who] has to find a place to sit down every time she comes into clinic”*

(P24)

Additionally, there are situations in which patients may not be comfortable having consultations with pharmacists in waiting areas where other staff and patients can overhear their conversation, as described by this outpatient pharmacist:

*“And most of the time they were very happy to have a chat out there, especially if I was seeing them before the doctors, they wouldn't miss the doctors, per se. But other times, there was a confidentiality and whatnot…”*

(P05)

### 7.3 Subtheme 3 Multi-disciplinary team communication breakdown

#### 7.3.1 A lack of communication between the hospital and community care setting

Interviewees described the issue of communication breakdowns between the hospital multi-disciplinary team and patients' primary care clinicians (GPs) who are central to patients' ongoing care. Clinic team members expressed concern that they assume that the GPs have received the updated discharge summary, but uncertainty exists as there is no follow up with the GP:

*“Yeah, definitely hard to connect to them [GPs] ...hard to comment how receptive they are because they're so hard to contact. … I mean I think a lot of people are aware that you need to bridge this gap and you need to just do things better”*

(P14)

Pharmacists explained how they were attempting to bridge this gap by communicating directly with patients' GPs. Pharmacists believed that patient care improved when they began co-ordinating transitions of care with GPs and educating patients about connecting with their GP. This interaction between pharmacists and the patients' GPs is important as minimising medication-related problems has been shown to prevent rehospitalisation and reduce the economic healthcare burden.^11^^,^^29^^,^^37^

#### 7.3.2 Working apart from clinicians interferes with effective/restricts communication

Pharmacists reported that working in an environment that does not allow in-person interaction with clinicians may interfere with open communication and lead to increased medication errors and reduced patient safety.

*“Unfortunately, we're not all well connected from a communication perspective …we are not all nearby, we are based at different sites. … What you want to avoid is, like a, you know, moving away or providing information that is like different to what someone else is trying to achieve”*

(P14)

See above – Clinic Staff Support – re: references about how in-person facilitates relationship building.

#### 7.3.3 Lack of role clarity

Unsurprisingly, there were reports of personality mismatches within the clinic teams as well as differences in perceptions and priorities related to the hierarchical structures in some clinics. Pharmacists in some clinics felt that a poor understanding of each other's professional roles within the healthcare team led to a misalignment of their goals which was further compounded by a negative team culture. These pharmacists were challenged to provide the patient care they believed was within their scope of practice, as illustrated in the following exemplar:

*“...try to integrate [a pharmacist into the clinic]… with people who traditionally don't know how to work with [a pharmacist] and don't really know how to refer a patient to…”*

(P14)

Fractured relationships with some clinicians may be built on an incorrect assumption that a pharmacist consultation simply impedes or delays the doctor's consult. Resultantly, pharmacists feel they must advertise their role to clinicians and repeatedly highlight what they can offer in terms of skills or expertise. The literature clearly shows how using the full scope of a pharmacist's capabilities enhances medication management pathways.^37^ This study showed that clinic teams who understand this were more receptive to the pharmacists.

“*So more for that chronic symptom burden, I definitely see the role of the pharmacist having the wisdom to guide, to guide what we try next…”*

(P16)

Consequently, it seems that a greater emphasis needs to be placed on understanding the importance of interprofessional collaboration and specifically on how to effectively integrating pharmacists on the healthcare team.^8^ Schindel and researchers also recognised that overlapping duties and responsibilities by different team members may cause role confusion and conflict. They concluded that educating patients and the healthcare team of the pharmacists' role may alleviate this problem.^28^

#### 7.3.4 Tunnel vision healthcare

Study pharmacists relayed concerns about the fragmented approach to healthcare that is not always collaborative. For example, interviewees noted that specialities within medicine often only prioritise and manage issues related to their field.

*“… sometimes we are time poor, and it's some there's lots of different pressures, but sort of fixing just the problem and letting a patient go, I think we're just almost using like a band aid approach”*

(P31)

This narrow healthcare focus as described by interviewees lacks patient-centredness, and has been shown to increase the risk of patient mismanagement and medication errors.^8^^,^^37^

### 7.4 Subtheme 4 Patient specific

#### 7.4.1 Patient fatigue

Some pharmacists reported that patients have reacted negatively to having an additional clinic consultation with a pharmacist as their perception is that they are unnecessarily required to stay at the clinic longer.

*“…the patients can get a bit frustrated with the amount of time that they have to spend seeing each person [health professional]. Occasionally I‘ll get a patient that'll be like ‘Why am I seeing pharmacy? I already saw the doctor!’ You know... ‘You've already asked me questions about my meds.’”*

(P04)

Outpatient pharmacists go on to explain that this situation is inaccurate as the pharmacist consult is typically slotted in between other consultations. However, it can be challenging for pharmacists to have an effective conversation with a patient about their medications when the patient perceives the pharmacist consult as an activity which lengthens their time at the hospital.

*“And I think sometimes they see, seeing the pharmacist as keeping them in clinic longer and then I have to try to explain to them that I'm just seeing them in this time before the, the doctors get to them. So I'm actually making better use of your time”*

(P19)

Planning for the appointment, early booking and establishing a rapport with the patient at the start of the series of consultations is important. In one Australian study, the doctors and pharmacists consulted in the clinic at the same time. This allowed a 3-way conversation about medications, reduced clinic time and promoted deprescribing (Thrive).^38^

## 8 Limitations

This was a single-site study and thus, the findings may not be generalisable or transferrable to some hospital clinic settings, as sites may be unique in their clinic activities, diversity and integration of healthcare team members, and locality (rural versus urban). Although interviews were conducted by a person independent of the study, interviewees may have felt obligated to provide socially and/or professionally desirable responses to the interview questions, thus introducing bias. Additionally, pharmacist participants were asked by the research team to identify clinic team members to participate in study. Thus, it is possible that interviewees over-reported the benefits, as they were aware the findings would be reported to the healthcare decision makers and possibly influence funding decisions. This study mainly reports the experiences of pharmacists, therefore, insights from other clinicians (i.e. doctors and nurses) are limited. Furthermore, participants being interviewed may have experienced time pressures (an identified theme) to get back to their clinic work which may have resulted in hasty or underreporting of challenges. However, the team focussed on having sufficient trustworthiness and rigor in the methodology, in particular the interview strategy and thematic analysis which was done by experienced pharmacists unknown to the hospital team.

## 9 Conclusion

This study explored pharmacists' and other health professionals' perspectives of the impact of having pharmacists working within interprofessional teams in hospital outpatient clinics. The strength of this study is that it is a novel exploration of pharmacists' and other health professionals' views of how they perceived the new outpatient pharmacist role in their clinics. These study findings provide detailed insight into participants perceived benefits of outpatient pharmacists to patient care, clinic teams, and healthcare systems as well as the challenges and contributors to successfully introducing pharmacists in outpatient clinics. Interviewees provided rich descriptions about the three overall themes that included the benefits, the contributors, and the challenges to introducing clinical pharmacy services to outpatient clinics. Benefits of having a pharmacist on the outpatient clinic team, as described by the team members, included continuity of patient care, improved medication management and safety, as well as a streamlining of the workflow through the clinic. Main contributors to the successful introduction included positive pharmacist attributes such as adaptability, which strengthened interprofessional relationships and facilitated communication. Factors that challenged or acted as barriers to the implementation of outpatient pharmacist positions included scheduling issues and a lack of physical space, multi-disciplinary communication breakdown, and role ambiguity. This can be addressed by having clear role descriptions, expectations outlined in advance and writing work guidelines. Future research could focus on the changes in clinical outcomes for patients seen within outpatient clinics that include clinical pharmacists^40^, ^41^, the impact of the coronavirus pandemic on the clinics^42^, as well as the implementation science and organisational elements that enabled the clinic roles to succeed.

## Author contributions

**Table:** 

Conceptualization	Dr Centaine Snoswell and A/Prof Michael Barras
Funding acquisition	Dr Centaine Snoswell
Ethics and governance	Dr Centaine Snoswell and A/Prof Michael Barras
Methodology	Dr Centaine Snoswell
Data Curation	Monica Taylor (please acknowledge) and Dr. Centaine Snoswell
Formal analysis	Amelia Cossart, Dr. Bernadette Chevalier, and Dr. Centaine Snoswell
Writing - Original Draft, and Writing - Review & Editing	Amelia Cossart, Dr. Bernadette Chevalier, A/Prof Michael Barras and Dr. Centaine Snoswell

## Funding source statement

The study is funded by an Early Career Researcher Grant awarded to C.S. from The University of Queensland.

## Declaration of Competing Interest

None to report.

## Appendix A Supplementary data

Supplementary material: Seeding interview questions Image 1
