# Supplementary material for: Benefits, challenges and contributors to the introduction of new hospital-based outpatient clinic pharmacist positions
Source: Explor Res Clin Soc Pharm. 2022 Feb 26;5:100119. doi: 10.1016/j.rcsop.2022.100119 (PMC9029912; doi:10.1016/j.rcsop.2022.100119)
Supplement: Supplementary file 1 — Supplementary material: Seeding interview questions [file mmc1.docx]

# Appendix A: Seeding interview questions

Q1) Please describe role of an outpatient clinic pharmacist in your clinic.

Prompts: What do they do for the clinic? What do they do for the patient? How do they fit into the team? How to they add to the patient journey?

What is the purpose and structure of the pharmacist consultations?

Q2) Can you describe some of the benefits, if any, of this/these role/s?

Prompts: To the clinic? To the patients?

Q3) What factors have contributed to this success or these benefits?

Q4) Can you describe some of the drawbacks, if any, of this/these role/s?

Prompts: To the clinic? To the patients?

Q5) What factors have contributed to these drawbacks?

Q4) What are some biggest challenges you have encountered as part of your role? (Pharmacists only)

Q6) Do you think the pharmacists directly impacted on services or patient outcomes?

Implementation

Q7) What factors made the implementation of this/these role/s possible?

Prompts: Collaboration? Partnership? Existing relationships? Financial agreements?
An existing business case?

Q8) What barriers were encountered during the implementation of this role/s?
Q9) Is there anything we have not discussed that you would like to add in regards to the outpatient clinic pharmacists at PAH?

Optional questions (potentially relevant managers only)

Q10) What would you consider are some of the biggest achievements of this/these position/s in your organisation? What do you think has enabled these successes?

Q11) What factors will facilitate ongoing success of this role?

Questions for pharmacists working in clinics (can be adapted for other clinic staff)

Q12) Can you describe how working in the clinic setting has affected your working relationship with other colleagues (Drs and nurses)?

Prompts: Give specific examples of scenarios where you have worked collaboratively

Q13) Can you describe what you typically do each day/ for each patient when working in your clinic role?

Prompts: Why do you do that? How easy/ challenging do you find this process?
